# Colloidal Shear-Thickening Fluids Using Variable Functional Star-Shaped Particles: A Molecular Dynamics Study

**DOI:** 10.3390/ma14226867

**Published:** 2021-11-14

**Authors:** Rofiques Salehin, Rong-Guang Xu, Stefanos Papanikolaou

**Affiliations:** 1 Department of Mechanical Engineering, Colorado State University, Fort Collins, CO 80523, USA; 2 Department of Mechanical and Aerospace Engineering, The George Washington University, Washington, DC 20052, USA; xrg117@gwmail.gwu.edu; 3 NOMATEN Centre of Excellence, National Centre of Nuclear Research, A. Soltana 7, 05-400 Otwock, Poland; stefanos.papanikolaou@ncbj.gov.pl

**Keywords:** molecular dynamics, functional particles, jamming, shear thickening, viscosity, diffusivity

## Abstract

Complex colloidal fluids, depending on constituent shapes and packing fractions, may have a wide range of shear-thinning and/or shear-thickening behaviors. An interesting way to transition between different types of such behavior is by infusing complex functional particles that can be manufactured using modern techniques such as 3D printing. In this paper, we perform 2D molecular dynamics simulations of such fluids with infused star-shaped functional particles, with a variable leg length and number of legs, as they are infused in a non-interacting fluid. We vary the packing fraction (ϕ) of the system, and for each different system, we apply shear at various strain rates, turning the fluid into a shear-thickened fluid and then, in jammed state, rising the apparent viscosity of the fluid and incipient stresses. We demonstrate the dependence of viscosity on the functional particles’ packing fraction and we show the role of shape and design dependence of the functional particles towards the transition to a shear-thickening fluid.

## 1. Introduction

The apparent viscosity of a fluid is an important component of various applications, such as the ones that include the control of mechanical vibrations or nanocoatings. In addition, the concept of a diverging viscosity with strain rate, has traditionally been under the basic theories of glasses, as they emerge in granular and colloidal systems [1]. Nevertheless, in practice, with common fluids and macroscopic components, using a variable viscosity concept might not be possible or would be difficult to implement in several cases. For this reason, magnetorheological (MR) [1,2] and electrorheological (ER) [3,4,5,6,7,8] fluids have emerged, which have the ability to control the fluid viscosity with external magnetic and electric fields, respectively. A continuous control of the viscosity would require additives, when using a common fluid [9,10,11] but without applying external force fields. For that reason, in this paper we focus on simulations of controlling the fluid viscosity [12,13,14,15,16,17,18] through infusing complex-shape functional particles [19,20,21,22] (like ferrous particles infused in MR or ER fluid) based on the concept of jamming [22,23,24,25,26,27,28,29,30] that takes place in randomly packed ensembles and therefore controls the damping characteristics [31,32,33] of generic fluids. The idea of a fluid viscosity increase lies in studies of jamming in particulate matter, in which jamming could occur as the packing fraction of matter increases beyond the jamming density, in frictional [28] or frictionless particles [34] interactions. However, in the presence of friction, the jamming characteristics and the viscosity increase are controlled and tuned by the friction coefficient. The geometric asperity (GA) model [22,29] demonstrates these effects through a two-dimensional (2D) model that has bidisperse disks having asperities centered at the disk edges, and the model considers asperity interactions that successfully emulate the effects of friction in particulate matter. Moreover, the GA model showed that instead of emulating physical friction, asperities can be tuned in a way that allows the control of fluid viscosity. In this paper, functional particles consist of disks that contain ray-like asperities throughout their surface and asperity collisions with the colloidal fluid are considered during stochastic rotation dynamics (SRD) runs. We find that the control of viscosity through these additive functional particles is quite a natural effect, and a natural extension of the results from the GA model [22].

**Figure 1:**
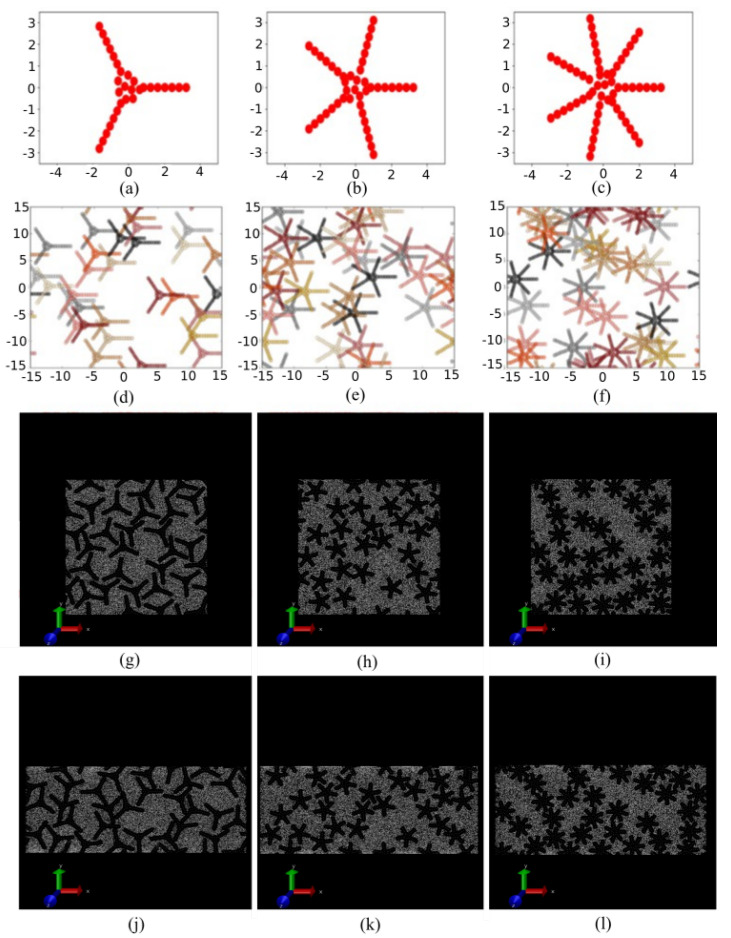
Functional particle configuration construction, for single (**a**) three, (**b**) five and (**c**) seven legs in a single star particle with core radius of 0.65 σ and leg length of 2.5 σ. Randomly dispersed configurations of 30 star particles in each system for (**d**) three, (**e**) five and (**f**) seven legs on each functional particle. Snapshot of MD simulation movies, before the pure shear applied on the system of star particles, with (**g**) three, (**h**) five and (**i**) seven legs infused in square-shaped (30 σ × 30 σ) periodic boxes filled with solvent SRD molecules and after the pure shear applied on the same system of star particles with (**j**) three, (**k**) five and (**l**) seven legs.

There are several existing approaches to study the rheology of particle suspensions in fluids. Among them, there are dissipative particle dynamics (DPD) [35,36], Brownian dynamics [37], lattice Boltzmann method [38,39], Stokesian dynamics [40,41,42] and SRD [18,43,44]. In order to take into account the temperature equilibration in an approximate but fast manner, in this paper, we utilize SRD, available in the simulation software LAMMPS [45,46]. The focus of this work involves the calculation of the fluid viscosity as the shape and packing fraction of the infused particulates are changing. Selecting particular particle shapes can provide a fine-tuning of the mechanical responses [47]. The emergent strength of packings made out of these functional particles depends on their shape, and they are stiffest/softest for compact and rod-like shape, respectively. The star-like functional particles in this work are formed through a custom algorithm that adds particulates stochastically towards a macroscopic target geometry (Figure 1) and the shape we choose is optimized so that it is stiffest at its center and softest at the outer sides. Strain stiffening happens due to soft packing at the outer side near jamming and the algorithm may be applied mutatis mutandis in three dimensions.

Soft rod-like shapes are often used in nanoparticles coating. Such coated nanoparticles have many applications in biomedical sciences, such as drug delivery, molecular imaging, biosensing, etc. Studies have been performed to understand the effect of coated surfaces with various patterns for nanoparticles on their effectiveness in such applications. For example, four different patterns, formed due to coatings using such line-shaped ligands around nanoparticles, were studied in [48]. The main result was that the identification of an optimal shaping among those four patterns required an anisotropic ligand pattern around the nanoparticle, in a way that enables the easy translocation of the coated nanoparticles. The dynamics of such penetration confirms the effect of surface patterning in such coated nanoparticles. Moreover, Bogart et al. reported that mixtures of multiple ligands are important for biocompatibility purposes [49], thus the possibility of jamming in such fluids may be quite high. In addition to such surface design in nanoparticles, underlying challenges exist, regarding the material characterization as well as the magnetism in nanoparticles. Such a material characterization for cases with gold nanoparticles allowed for a better membrane penetration [50]. Such nanoparticles could provide a basis for colloidal fluids such as the ones investigated in the current work.

The main focus of this work is the demonstration of the shear-thickening behavior of colloidal fluids with infused functional particles [25]. The leg length and number of legs both determine the packing fraction in the fluid, and these can be varied by the algorithm that is used for particle generation. Due to jamming, the system transforms from liquid-like state to a shear-thickened, and then to a rigid, disordered, solid-like state. Other shapes, spherical or circular [51,52], ellipsoidal [53,54,55], rods [56,57,58], polyhedra [59] and U-shaped [25] particles have already been studied to investigate jamming effects. However, the effects of more complex-shape particles, in relation to jamming, remain an interesting open question.

In this paper, we aim to perform a proof-of-principle demonstration, so we utilize two-dimensional molecular dynamics simulations using LAMMPS [45,46], performed by infusing star-shaped particles with various numbers of legs and leg lengths into particles that act as background fluids. Our principal focus is the qualitative demonstration of the effects that may then be pursued in the laboratory. We perform an analysis of the shear stress and viscosity with respect to strain rates, to find that the liquid-like state is transforming into a solid-like state with a drastic increase of the apparent fluid viscosity. From the behaviors of pressure vs. packing density and viscosity vs. strain rate, we identify approximate jamming packing fractions, where the transition from shear thickening to jamming takes place. The higher the packing fraction, the higher the total strain and time required to reach a steady state. Most importantly we show the existence of a jump of viscosity after reaching the jamming transition, for distinct functional particle shapes, and associated jamming effects. Finally, the structure of the system is analyzed and the main results include the features of particle diffusion in a shear-thickening fluid, and the features of the primary peaks in the microstructure’s pair correlation function. The rest of the paper is organized as follows. In Section 2, the molecular model and simulation method will be described in detail. In Section 3, we present detailed MD simulation results and a discussion, followed by our summary in Section 4.

## 2. Materials and Methods

In this work, we used two-dimensional periodic simulations in LAMMPS with side length of 30 in LJ units [60]. This unit is widely used in MD simulations for reporting generalized results that can be interpreted into physical units for any specific materials [44]. We chose SRD particles that act as the background solvent for rigid colloidal star particles. In our MD simulations, the density of SRD particles (solvent) was set at 85.0 in LJ units. The density of star particles varied since the simulation cell can be filled with different size star particles. The mass of each SRD atom was set at 1.0 and the mass of each star particle atom was set at 100, which aimed to approach the limit of infinite relative mass ratio, with functional particles having ideal collisions with SRD particles, and was consistent with previous publications [61,62,63,64]. As an example of relative density of star particles, in our simulations, for a simulation cell infused with a 3-legged star particle that has a leg length of 3.0, the functional star-particle density was 106.67 in LJ units. We used a custom packing algorithm to generate the functional star particles using small circular core particles each having a radius *r* = 0.2. The overall packing algorithm contains two steps: in the first step, we perform 1000 trials to place core particles randomly in a circular disk to create the core of star-shaped particles with a radius R = 0.65 that rejects particles that are overlapping or are too close. Then, legs are placed equidistantly around the R-circle with an angle (θ) using the formula $\theta =2\pi /N_{leg}$, where $N_{leg}$ is the number of star-legs (i.e., 3, 5 or 7). The number of core particles in each leg (*n*) is an integer number determined by the formula $n=(L_{g}/\left(2r\right)+1)$ where Lg is the desired leg length. The radial distances are measured by rx=((R+r)cos(iθ) and new particles are added at a distance rx${}_{a}$ using the formula

(1) $$rx_{a}=\left(rx+j2r\cos\left(i\theta \right)\right)$$

that gives the variable leg length (L = 0.0 to 6.0 for 3 legs, L = 0.0 to 3.0 for 5 legs, L = 0.0 to 2.5 for 7 legs) as showed in Figure 1a–c. In the second step, 30 star particles with the same number of legs and each leg with the same length created by this exact same way are randomly dispersed in the square-shaped simulation box, allowing the overlap of star particles as in Figure 1d–f. The packing fraction (ϕ) for each different system is calculated by the area covered by the total number of small circular particles over the area of the square where the particles are dispersed, by the formula $\phi =(N\cdot \pi \cdot r^{2}\cdot /L^{2}$) where *N* is the total number of small particles and *L* is the side length of the simulation box [25].

SRD particles are coarse-grained background solvent constituents consisting of ideal, point particles. The detailed SRD formalism is described by Hecht et al. [63]. The essential idea behind using SRD particles as a cheap coarse-grained solvent is that SRD particles do not interact with each other, but only with the solute particles, i.e., the different shaped colloidal particles in this study. The fluid-like property (such as an effective viscosity) is generated by the collision and rotation properties of these point SRD particles, through particle streaming and particle velocity updates. Thus, simulations with large solute particles can be run more quickly and the solute properties such as viscosity and diffusivity in a background fluid can be measured.In our study, and in the limit of infinite relative mass ratio, the SRD approximation becomes quite accurate. In LAMMPS, the algorithm of SRD includes two steps: (1) the streaming step, in which it advects SRD particles, performs collisions between the SRD particles and the large solute particles, and thus imparts forces and torques to the large solute particles, which also change the positions and momentums of the SRD particles; (2) the velocity update step, in which it periodically resets the velocity distribution of the SRD particles via random rotations. The fundamental relation between the mass of SRD particles ($m_{SRD}$), temperature (*T*), characteristic time step (Δ*t*) and mean free path (λ) between collisions are related through the following relation λ = Δ*t*$\sqrt{k_{B}\cdot T/m_{SRD}}$. During the streaming step the $i^{th}$ particles position at time n+1 is calculated using an Euler scheme, $r_{i}^{n+1}=r_{i}^{n}+v_{i}^{n}\cdot \Delta t$, where Δt is the SRD time step that equals to the simulation time step multiplied by the number of time steps after which the SRD particle velocities reset. The particle velocity is updated using a rotation matrix $R[\xi \left(r_{i}^{n+1}\right)]$ with [63],

(2) $$v_{i}^{n+1}=v_{i}^{n}+R\left[\xi \left(r_{i}^{n+1}\right)\right]\left(v_{i}^{n}-u\left[\xi \left(r_{i}^{n+1}\right)\right]\right)$$

where $u\left(\xi ,t\right)=\sum_{k\epsilon \xi }v_{k}/N_{\xi ,t}$ is the average velocity of bin ξ, and $N_{\xi ,t}$ is the total number of SRD particles at time *t* [64]. In this study, the tangential component of the SRD particle momentum is preserved. Furthermore, we prevent the overlapping of star particles by setting the “overlap” tag to no.

For the simulations, we use a soft potential for star–star as well as star–SRD particles interaction. It is a repulsive potential that repels overlapping atoms and also becomes valid as *r* goes to 0. The potential is defined as,

(3) $$D_{it}=\left\{\begin{array}{cc} A[1+Cos\left(\pi \cdot r/r_{c}\right)] & \mathrm{when}\;r<r_{c}\\ 0 & \mathrm{when}\;r>r_{c} \end{array}\right.$$

where *A* is a typical pre-factor and $r_{c}$ is the cut-off (σ). In the simulations, we used the prefactor to ramp up from 0 to 100 towards configuration equilibration, and kept the global cutoff equal to 1.12 σ and the local cutoff equal to 1.0 σ. After the completion of the equilibration run, the prefactor remains at its final value during shear loading.

The key aspect of this work is the investigation of the shear-induced effects on fluid viscosity. The system is sheared for different strain rates starting from 0.001 to 0.009 in steps of 0.001 and 0.01 to 0.09 in steps of 0.01. Figure 1g–l show the system before and after shear, displaying the noticeable change in box dimensions. The negative pressure tensor in the diagonal gives the normal stresses. Shear stress is then calculated as follows [65]:

(4) $$\tau_{ij}^{s}=\sigma_{ij}-1/2\delta_{ij}\sum_{i,j}\sigma_{ij}$$

where $\delta_{ij}$ is the unit tensor being 1 if i=j and 0 if i≠j. The viscosity is related to the shear stress and shear strain at point *p* and time *t* as follows [66]:

(5) $$\tau^{s}\left(p,t\right)=\eta^{s}\dot{\gamma }\left(p,t\right)$$

where the total pressure is $P=(P_{xx}+P_{yy})/2$ and the fluid viscosity is then calculated from the slope of the shear stress vs. strain rate plot as the strain rate approaches zero.

For the diffusion coefficients, we calculate the mean square displacement (MSD) of a group of atoms. For calculating the diffusion coefficients of molecules (star particles composed of several number of relatively fixed position atoms) we calculate the center of mass for each molecule and then consider that the center of atoms has a MSD [67]

(6) $$MSD=\bar{{\left(r-r_{0}\right)}^{2}}=1/N\sum_{n=1}^{N}{\left(r_{n}\left(t\right)-r_{n}\left(0\right)\right)}^{2}$$

where *N* is the number of total atoms, $r_{n}\left(0\right)=r_{0}$ and $r_{n}\left(t\right)$ is the initial position and the position after time *t* of atoms, respectively. The radial distribution function, g(r), shows the structure of the suspended star particles with different shapes. The g(r) is calculated for different initial conditions of same leg length and number of legs.

## 3. Results

### 3.1. Shear Thickening Due to Functional Particle Infusion

**Figure 2:**
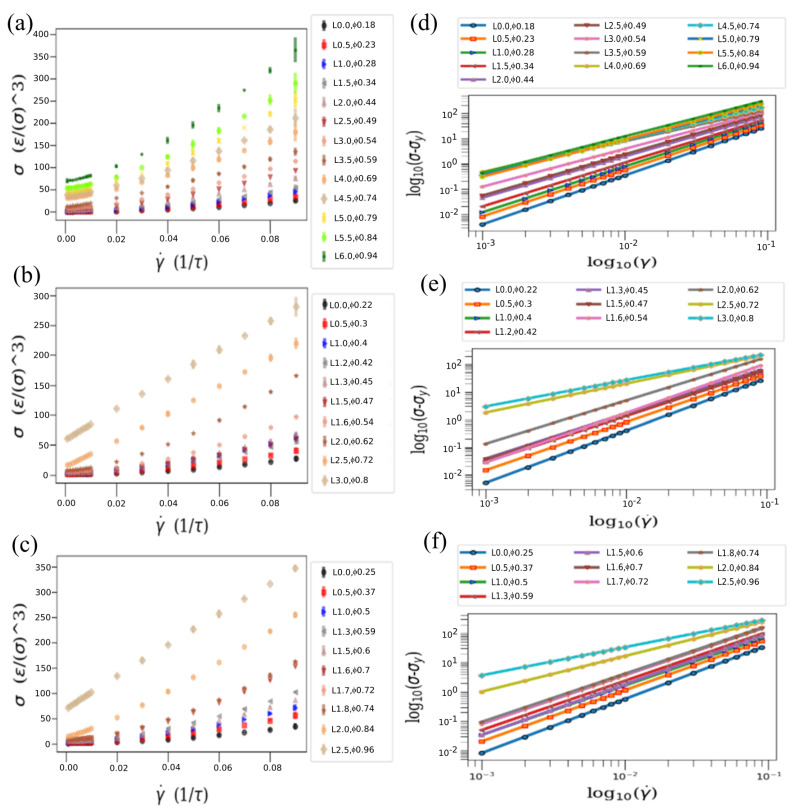
Fitting plots of shear stress ($\epsilon /\sigma^{3}$) vs. strain rate (1/τ) for: (**a**) 3-legged star particles with packing fraction, ϕ, of 0.18, 0.23, 0.28, 0.34, 0.44, 0.49, 0.54, 0.59, 0.69, 0.74, 0.79, 0.84 and 0.94; (**b**) 5-legged star particles with packing fraction, ϕ, of 0.22, 0.30, 0.40, 0.42, 0.45, 0.47, 0.54, 0.62, 0.72 and 0.80; (**c**) 7-legged star particles with packing fraction, ϕ, of 0.25, 0.37, 0.50, 0.59, 0.60, 0.70, 0.72, 0.74, 0.84 and 0.96. The log–log plot of shear stresses normalized by removing the respective yield stresses with respect to the strain rates for (**d**) 3-legged, (**e**) 5-legged and (**f**) 7-legged star particles.

A key effect of the presence of the functional particles in the fluids is in the response to applied shear strain rates. At small strains, the overall stress is close to zero and represents a zero-shear viscosity state. However, an increase in the strain rate can nonlinearly increase the stress leading to sufficiently high values. Figure 2a–c indeed shows that, the slope of the stress vs. strain plot is zero near a very low strain rate (0.001). However, the gradual increase in the strain rates up to a strain rate of 0.09 shows that the slope reaches a comparatively larger value than the slope at lower strain rates. This shows that, the shear stress increases more compared to an increase in strain rate. In addition, we observe that the leg lengths are also important in this viscosity increment. For a system with three-legged star-particles, if the leg length is small (0.0–1.5) the statement of having zero shear stress at nominal stress is true. However, for the same strain rate, if the leg lengths are larger (6.0), we see an observable shear stress even at zero strain rate. This is even more noticeable if we consider the five-legged and seven-legged star particles with large leg lengths.

A standard way of investigating the flow stress of such complex fluids is through the fitting of the stress–strain-rate relationship. Because of a nonlinear increase in shear stress caused by infusing star-shaped particles in fluid-like SRD, we can determine the gradual rise of yield stresses in our system by using a non-Newtonian fluid model [19,68] of the form:

(7) $$\sigma =A{\dot{\gamma }}^{\alpha }+\sigma_{y}$$

where *A* is the slope, α is the exponent and $\sigma_{y}$ is the yield stress. Figure 2d–f shows the yield stresses for three-legged, five-legged and seven-legged star particles with different leg lengths that vary the overall packing fractions. When α is less than 1, a shear-thinning behavior is observed, while for α>1 a shear thickening is expected.

In the model we studied, the fluid is Newtonian for small packing fractions, but develops a yield stress as the packing fraction increases, similar to several previous works [65,69,70,71,72], as seen in Figure 2a–c. One can see the rise of solid-like properties in those fluidic systems. At the same time, these plots show the increase towards a shear-thickening fluid for large packing fractions and large number of legs. The same conclusion can be made from Figure 2d–f where a log–log plot of normalized shear stresses are plotted against strain rates. The slope of the plot indicates how much net stress above yield stress there is for different systems that contain different packing densities of infused star particles with respect to different strain rates. We find from these that the normalized shear stress is positive for a system with higher packing density even at a lower strain rate and the stress sees further increment for an increment of strain rate.

**Figure 3:**
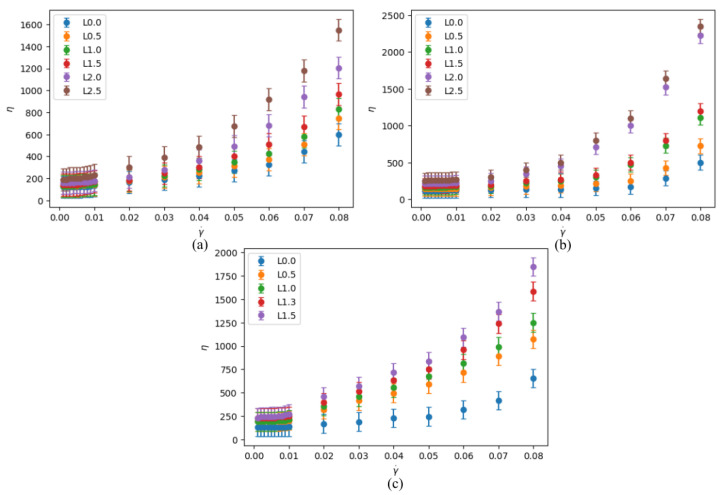
Viscosity ($\epsilon \tau /\sigma^{3}$) vs. strain rate (1/τ) with leg length L (σ) of: (**a**) 0.0 σ, 0.5 σ, 1.0 σ, 1.5 σ, 2.0 σ and 2.5 σ for 3-legged star particles; (**b**) 0.0 σ, 0.5 σ, 1.0 σ, 1.2 σ, 1.3 σ and 1.5 σ for 5-legged star particles; (**c**) 0.0 σ, 0.5 σ, 1.0 σ, 1.3 σ and 1.5 σ for 7-legged star particles.

To demonstrate the fluid-like to solid-like transition in a jammed state, we also determined how the viscosity varied with strain rate for various packing fractions and numbers of legs. Figure 3a–c shows that, with an increase of strain rate the viscosity also increases, which agrees with our previous result from Figure 2a–c, where we demonstrate that shear stresses increase with strain rates. The increase of viscosity raises the solid-like properties in our simulated system. This increase of viscosity makes our fluid of interest a characteristic shear-thickening fluid. The shear-thickening behavior is similar for all the three-legged, five-legged and seven-legged star particle system as expected. However, the magnitude of the viscosity is observed to be different in different systems where particle shapes are different. Characteristically, the fluid becomes deeply shear thickening as the number and/or the length of the legs increase.

### 3.2. Pressure, Viscosity, Diffusivity and the Onset of Jamming

**Figure 4:**
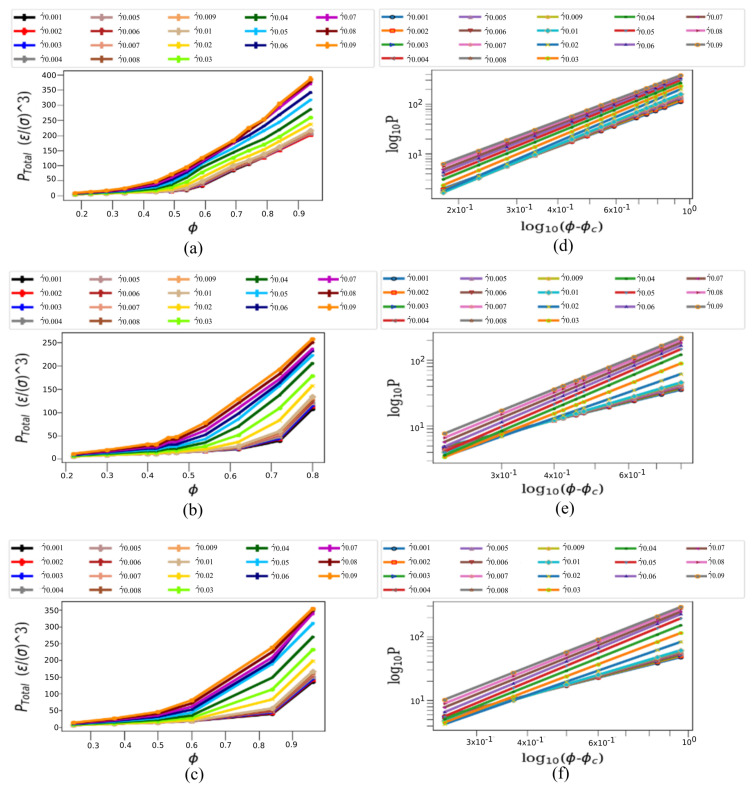
Total Pressure and Packing Fraction. (**a**) Total pressure ($\epsilon /\sigma^{3}$) vs. packing fraction (ϕ) for strain rates equal to 0.001, 0.002, 0.003, 0.004, 0.005, 0.006, 0.007, 0.008, 0.009, 0.01, 0.02, 0.03, 0.04, 0.05, 0.06, 0.07, 0.08 and 0.09 for 3-legged star particles, and with ϕ equal 0.18, 0.23, 0.28, 0.34, 0.44, 0.49, 0.54, 0.59, 0.69, 0.74, 0.79, 0.84 and 0.94. (**b**) $\epsilon /\sigma^{3}$ vs. ϕ for same strain-rates as (**a**), for 5-legged star particles with ϕ equals 0.22, 0.30, 0.40, 0.42, 0.45, 0.47, 0.54, 0.62, 0.72 and 0.80. (**c**) $\epsilon /\sigma^{3}$ vs. ϕ for same strain-rates as (**a**), for 7-legged star particles with ϕ equals 0.25, 0.37, 0.50, 0.59, 0.60, 0.84 and 0.96. (**d**–**f**) The total pressure is shown as function of $\phi -\phi_{c}$ in log-scale, in correspondence to (**a**–**c**).

A way to investigate the jamming onset in the fluid arises through the tracking of the steady-state average of the total pressure as a function of the packing fraction. At a strain rate close to zero the total pressure represents the pressure along the yield stress curve [25]. In principle, we expect to see *P* vanish near the jamming packing density and then rise to a finite value above jamming. In Figure 4a–c we find that the pressure begins to converge and then diverges to finite values for packing fraction (ϕ) between 0.5 and 0.6 for systems infused with three-, five- and seven-legged star particles. As it is well known [22,29], it is actually difficult to locate the exact jamming packing density value from tracking features in the curve as a simulation system size scaling is mandatory, and a critical scaling analysis is necessary to determine the jamming transition [17,73,74]. By using characteristic power-law fitting forms $P=B{\left(\phi -\phi_{c}\right)}^{\beta }$ when $\phi >\phi_{c}$ and P=0 when $\phi \leq \phi_{c}$, where *B* is the slope, we estimate β as the power and we identify the jamming packing fraction $\phi_{c}$ to demonstrate the non-monotonic evolution with respect to the strain rate. The power is always greater than 1 for all configurations, indicating a shear-thickening behavior. The jamming packing fraction increases with respect to the increase in strain rate for five-legged and seven-legged suspensions. Finally, another way to look at quasi-static behavior and determine the jamming packing fraction is to investigate the pressure vs. strain rate behavior, for a fixed packing fraction. For $\phi <\phi_{c}$, we get the $P/\dot{\gamma }$ value to a finite value as $\dot{\gamma }$ reaches zero and for $\phi >\phi_{c}$ we find that $P/\dot{\gamma }$ diverges as $\dot{\gamma }$ reaches zero. In this case, we find a lower bound for $\phi_{c}$ = 0.54 for three-legged (Figure 4a), $\phi_{c}$ = 0.54 for five-legged particles (Figure 4b) and $\phi_{c}$ = 0.6 for seven-legged ones (Figure 4c).

**Figure 5:**
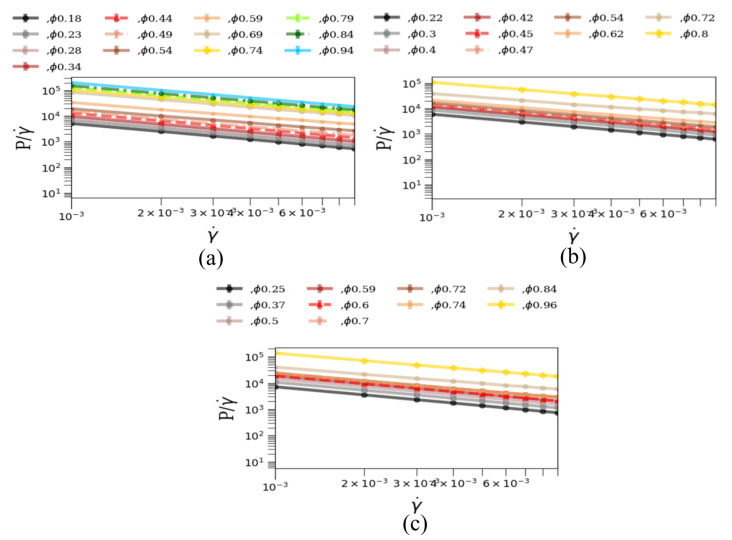
Pressure to strain rate ($\epsilon \tau /\sigma^{3}$) vs. strain rate (1/τ) plot for system infused with (**a**) 3-legged, (**b**) 5-legged and (**c**) 7-legged star particles.

**Figure 6:**
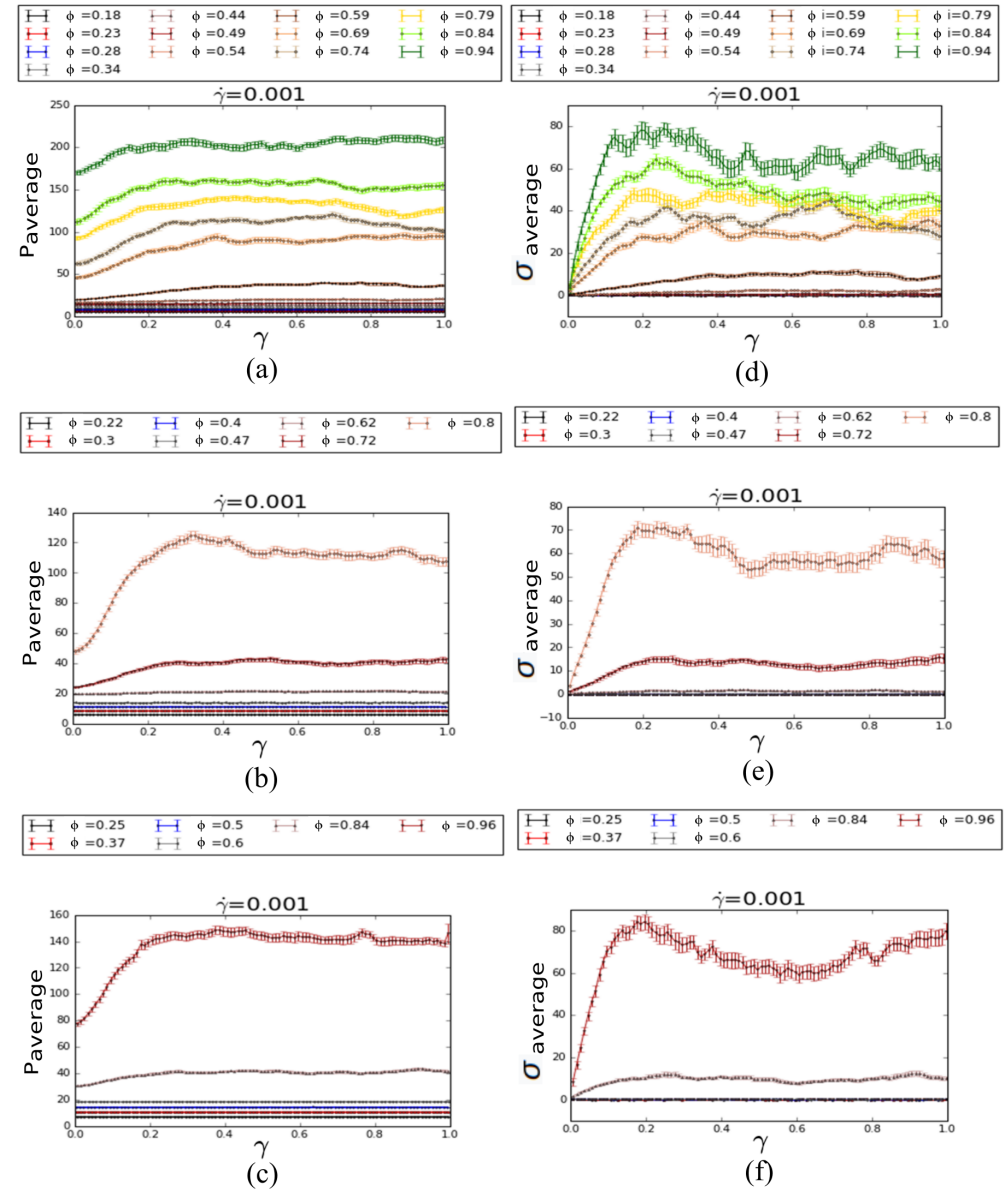
Average total pressure ($\epsilon /\sigma^{3}$) and shear stress ($\epsilon /\sigma^{3}$) vs. strain for constant strain rate (1/τ) of 0.001 for: (**a**,**d**) 3-legged star particles with packing fraction ϕ of 0.18, 0.23, 0.28, 0.34, 0.44, 0.49, 0.54, 0.59, 0.69, 0.74, 0.84 and 0.94; (**b**,**e**) 5-legged star particles with packing fraction ϕ of 0.22, 0.30, 0.40, 0.47, 0.62, 0.72 and 0.80; (**c**,**f**) 7-legged star particles with packing fraction ϕ of 0.25, 0.37, 0.50, 0.60, 0.84 and 0.96, with standard error of the mean error bars. Each point of the plots represents the (**a**–**c**) average of pressure and (**d**–**f**) average of shear stress over a strain window Δt = 1000. The non-monotonic behavior seen at very high packing fractions is a direct evidence of yielding behavior from the jammed state of the hybrid fluid/functional particles system.

For each particular system we applied uniform strain rates starting from 0.001/s to 0.09/s. In Figure 5, the overall total pressure is shown with respect to the applied strain-rate for the 3 topologically distinct fluids (functional particles with 3, 5 or 7 legs (*cf.* Figure 5a–c respectively)). As expected, the total pressure scales linearly with the strain-rate for all cases, due to the repulsion of the functional particles, as they move at higher rates. Figure 6a–c and Figure 6d–f one can see the average pressure and average shear stress, respectively, as a function of net shear strain for several different packing fraction (ϕ = 0.18, 0.23, 0.28, 0.34, 0.44, 0.49, 0.59, 0.69, 0.84 and 0.94 for 3-legged; ϕ = 0.22, 0.30, 0.40, 0.47, 0.62, 0.72 and 0.80 for 5-legged; and ϕ = 0.25, 0.37, 0.50, 0.60, 0.84 and 0.96 for 7-legged star particles) for a uniform strain rate of 0.001/s. Each pressure and shear stress point in the plot represents the local average of pressure and shear stress over a strain window of Δ t = 1000. These plots have error bars as standard errors of mean over 30 different initial configurations for each packing density. From these plots we can clearly see that after a certain total strain value all the systems reach a steady state as we expected earlier. However, for a higher packing fraction, both the relaxation time and total strain values are much higher than at a lower packing fraction, for each type of star particles, indicating the glassiness features expected for such frustrated fluids. Furthermore, it is clear that for high packing fractions, apart from the increase of stress, the stress becomes non-monotonic, a direct evidence of plastic yielding from a jammed state, formed by the packed functional particles and the fluid.

Further, the average viscosity was investigated for each leg length with 30 different initial condition from the average of slope for the first six and eight points of shear stress vs. strain rate plots. This average result eliminates the initial condition dependence as well as any specific factor that may lead to the jamming state. The viscosity is nearly zero until the packing fraction reaches 0.5, for systems with five-legged and seven-legged star particles, whereas it is 0.6 for system with three-legged star-particles. The similar jump behavior can be explained in terms of the leg length of these particles. When the leg length of five-legged and seven-legged particles reaches 1.5 and that of the three-legged particles reaches 2.5, the viscosity rises significantly due to jamming. The jamming reaches a critical value with a higher packing fraction as well as leg length because of a less compact shape of the three-legged particles compared to the five-legged and seven-legged particles. This sharp increase of viscosity is the key indicator that the transition from liquid-like state to solid-like state is caused by the jamming of star particles.

**Figure 7:**
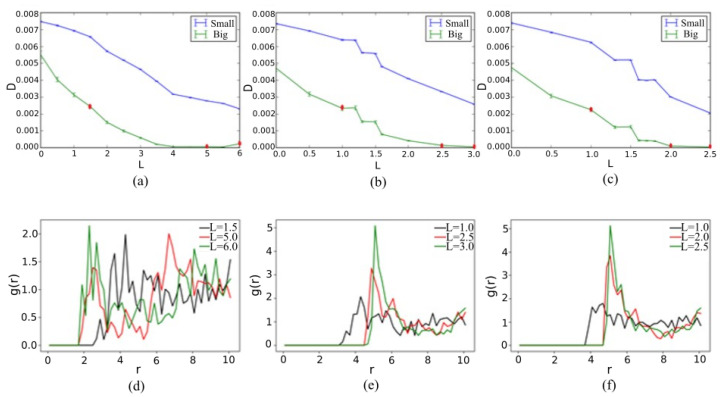
Diffusivity D ($\sigma^{2}/\tau$) of small SRD fluid and big star particles vs. leg length L (σ) of (**a**) 3-legged, (**b**) 5-legged and (**c**) 7-legged star particles system; radial distribution function g(r) of big star particles center of mass for radial increment of 0.2 σ and maximum radial distance 10 σ in: (**d**) average of all 30 configurations for 3 legs with maximum leg length L = 1.5 σ, L = 5.0 σ and L = 6.0 σ; (**e**) average of all 30 configurations for 5 legs with maximum leg length L = 1.0 σ, L = 2.5 σ and L = 3.0 σ; (**f**) average of all 30 configurations for 7 legs with maximum leg length L = 1.0 σ, L = 2.0 σ and L = 2.5 σ.

It is worth noting that the star particle shape has a direct effect on diffusivity. Diffusivity is calculated from the slope of the mean square displacements for small SRD atoms and big star particles’ center of mass as described in the methodology section. Figure 7a–c shows that star particles with longer legs have a high packing fraction and more inability to disperse freely without colliding and jamming with other star particles. The lower the arm length, the less restrictions for free movement, which results in higher diffusion of the functional star particles. The change in suspension structure for different shaped star particles and packing fractions can be visualized by plotting the radial distributive function g(r). The center of mass for star particles is considered and plotted for three-, five- and seven-legged star particles in Figure 7d–f. For three-legged star particles, the greater number of distinct peaks indicates a highly ordered structure, but it reduces into larger primary peaks as the arm length increases and the system gets more jammed. Similarly, for five-legged (Figure 7e) and seven-legged (Figure 7f) the primary peaks become larger and local ordering emerges as the arm length increases.

**Figure 8:**
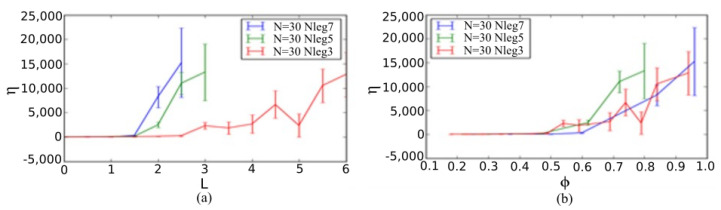
Viscosity ($\epsilon \tau /\sigma^{3}$) of the SRD fluids with respect to the (**a**) leg length L (σ) and (**b**) packing fraction ϕ of infused star particles. The number of star particles N = 30 and number of legs $N_{leg}=$ 3, 5 and 7 for star particles is indicated with red, green and blue color, respectively. It is worth noting that the viscosity can increase by multiple orders of magnitude, turning water-like fluids into gel-like texture by altering the functional particle shapes. Furthermore, for a given functional particle shape, the viscosity displays a divergence that clearly violates typical colloidal suspension rules, such as the Einstein formula for effective viscosity of a solvent with solutes $\mu_{eff}=\mu_{0}\left(1+B\phi \right)$, where $\mu_{0}$ is the solvent viscosity, ϕ the solute packing fraction and *B* a finite geometrical factor [75].

**Figure 9:**
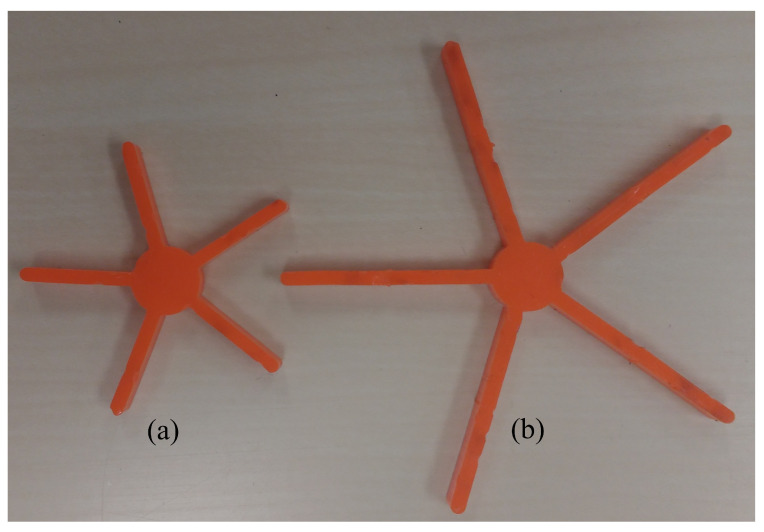
Five-legged star particles with core radius of 0.65 cm and leg lengths of (**a**) 1.5 cm and (**b**) 3.0 cm, created by laser cutting on Plexiglas.

Based on the results of this computational study, we can infer that these functional star-shaped particles have potential applications in ongoing rheology studies. Therefore, experimentally studying the effect of functional star-shaped particles on fluid viscosity would first require the feasibility and demonstration of expected effects and second, the creation of functional star particles using a form of particulate matter. On the former, as shown in Figure 8a,b, fluid viscosity may dramatically increase by increase the packing fraction ϕ beyond the jamming packing fraction $\phi_{c}$ (see Figure 8b), which can be achieved in a functional manner by increasing the leg length for any number of particles (see for example, Figure 8a). On the latter, while nanoscale investigations are certainly of great interest, their applicability extends to any scale; thus, we have created five-legged star particles by applying a laser cutting technique on Plexiglas sheets, to demonstrate a physical star-shaped particle, since the rest of the experimental study is beyond the scope of our current computational study at the macroscale. Figure 9 shows two of the five-legged star particles created by laser cutting at centimeter-dimensions. The core radius of each of the star particles is 0.65 cm and the leg length of the smaller and larger star particles are 1.5 cm and 3.0 cm, respectively.

## 4. Discussion

Colloidal suspensions have a long history. Standard equations exist, such as the Einstein equation for effective viscosity of a solvent with solutes $\mu_{eff}=\mu_{0}\left(1+B\phi \right)$, where $\mu_{0}$ is the solvent viscosity, ϕ the solute packing fraction and *B* a finite geometrical factor. However, the viscosity alteration due to such solute additives is limited. However, a novel pathway exists by engineering design of functional particles that contain an intrinsically collective mechanism for shear thickening and jamming [22]. Through such a design, in this work, we investigated infused star particles in two dimensional simulations, as proof-of-principle, for the shear-thickening and jamming effects demonstration. Indeed, functional star particles caused shear thickening and jamming in simulated SRD fluids. Shear thickening could be easily identified by having a nonlinear stress–strain-rate response at low packing fractions. Moreover, we identified the key features related to jamming, to draw conclusions related to the shear stresses, pressure, viscosity and diffusivity of our system, which vary with applied strain rates as well as the packing fraction of the infused functional star particles. It is worth noting that the viscosity can increase by multiple orders of magnitude, turning water-like fluids into gel-like texture by altering the functional particle shapes [75]. Furthermore, for a given functional particle shape, the viscosity displays a divergence that clearly violates typical colloidal suspension rules, such as the Einstein formula for effective viscosity. The observation of shear stress is also very important to identify the shifting of our fluid-like system towards shear-thickening behavior. Our results of stress with respect to strain rate clearly shows the increase of shear stress with respect to strain rate that leads to an increase of the yield stress of the incipient amorphous functional star particle structure. By observing the non-monotonicity of the stress as function of the strain, we inferred the presence of an amorphous glass/gel structure at high packing fractions, as expected from prior works [29]. The pressure and jamming analysis clearly indicated a jammed state caused by the infused star particles at a critical packing density, identified through pressure and viscosity divergences. The solid-like property showed an increase after this jammed state was achieved in different types of systems, i.e., three-legged, five-legged and seven-legged star particle systems. The key finding of our work is the rise of the viscosity with respect to the packing fraction of infused star-shaped functional particles. The viscosity showed a rise after the packing fraction reached 0.5 for five-legged and seven-legged star particles, and 0.6 for three-legged star particles. This showed the shape dependency towards a rise of the viscosity due to the jamming transition. The three-legged star particles are a comparatively less complex structure than the five-legged and seven-legged star particles. Therefore, the viscosity rose at a lower packing fraction for a comparatively complex shaped particles. Finally, the diffusivity of particles showed that the diffusion was consistent with shear-thickening fluid characters. The particle size was analyzed in this study, and we found that small fluidic particles were more diffusive compared to big star particles at any packing density. The probability of finding the star particles increased with a reduction of the diffusivity.

## 5. Conclusions

In this study, we demonstrated, by using MD simulations, how shear thickening takes place in systems of fluid-like SRD particles with infused star-shaped functional particles and variable leg numbers as well as leg lengths. We studied the limit of very massive functional particles (relative mass ratio of 100) and only in two dimensions, so that we could demonstrate a proof-of-principle calculation of the overall behavior in such fluids. For various strain rates, we found that functional star particles may cause a smooth Newtonian fluid to transform into a shear-thickened colloidal fluid and then to a jammed amorphous solid that yields analogously to a gel [75]. Shear thickening was identified by the nonlinearity in the stress vs. strain-rate response, seen at low packing fractions in a clear manner. Jamming took place after the packing fraction where pressure and effective viscosity displayed a divergence, and this was the critical packing fraction that ranged from 0.5 to 0.6 for different shaped star particles. We observed that the system reached an equilibrium after straining at a constant strain rate for a sufficiently long time, by studying the pressure of the system at constant strain rate. The larger the packing density or leg lengths, the more time was required. The diffusivity of big star particles as well as small fluid-like SRD particles also showed analogous effects. Jamming caused a reduction in diffusivity which was ordered in less compact, shaped three-legged particles and disordered in more compact, shaped five-legged and seven-legged particles. The dependence of the viscosity on the packing fraction shows that there is a very strong divergence that goes beyond typical colloidal suspension rules [75], and also provides a novel pathway for manufacturing functionally viscous fluids. Future work shall focus on experimental realization of such functional fluids for nanocoating applications.

## Data Availability

The data presented in this study are available on request from the corresponding author. The data are not publicly available due to privacy reasons.

## Abbreviations

The following abbreviations are used in this manuscript:

MDPI	Multidisciplinary Digital Publishing Institute
MR	Magnetorheological
ER	Electrorheological
SRD	Stochastic rotation dynamics
DPD	Dissipative particle dynamics
LAMMPS	Large-scale Atomic/Molecular Massively Parallel Simulator
GA	Geometric asperity
